# Locally advanced breast cancer in Brazil: current status and future perspectives

**DOI:** 10.3332/ecancer.2019.895

**Published:** 2019-01-22

**Authors:** Gustavo Werutsky, Paulo Nunes, Carlos Barrios

**Affiliations:** 1 Latin American Cooperative Oncology Group, Porto Alegre 90619-900, Brazil; 2 Department of Medical Oncology, Hospital São Lucas PUCRS, Porto Alegre 90619-900, Brazil

**Keywords:** breast cancer, treatment, survival, Brazil

## Abstract

Breast cancer (BC) is the most frequent cancer and the main cause of cancer deaths among women worldwide and in Brazil. A high proportion of patients are diagnosed with locally advanced breast cancer (LABC) in Brazil, mainly due to limited coverage of screening programmes. A disparity in the access to optimal treatment is evident between the public and private health systems which impact patient outcomes. Clinical research is an opportunity for patients, institutions and investigators and therefore should be facilitated through a better regulatory environment. In a country facing a trend of increasing BC incidence for the next years, it is critical to improve BC screening and incorporate new medicines and devices into the public health system to control the burden of LABC.

## Introduction

Breast cancer (BC) is the most frequent cancer and the main cause of cancer deaths among women worldwide and in Brazil. The World Health Organization estimated that 1.67 million new BC cases were diagnosed in 2012 (25% of all cancers) [1]. Data from the Brazilian National Cancer Institute estimate 59,700 new BC cases in 2018; therefore, BC is the most frequent neoplasm in women in almost every Brazilian region, excluding non-melanoma skin cancer [2]. The Global Burden of Disease Study [3] estimated 545,589 deaths from BC worldwide and 17,018 deaths in Brazil in 2016.

In the last 20 years, the incidence and death rates of BC have increased in Brazil. In the period from 2006 to 2016, the incidence rate had a 2.54% annual increase [3], whereas the death rate had a 12.2% increase from 1990 to 2015 [4]. In contrary, a decrease in BC mortality has been observed among most high-income countries of Europe and North America [5]. Brazil, similar to other countries in Latin America, is facing a demographic and epidemiological shift and consequently, a higher incidence of noncommunicable diseases, with cancer being one of the most prevalent diseases. Even though regional disparities inside the country are perceived in terms of diagnosis, treatment and outcome are associated with socioeconomic factors for example [6, 7].

## Locally advanced breast cancer (LABC) diagnosis in Brazil

### BC screening

Brazilian national guidelines published in 2015 recommend mammography screening at least every 2 years for women aged 50–69 years old and every year starting at 35 years of age for women with a family history of BC [8]. However, access to screening scans is a current issue in the country. The exact numbers of women that undergo screening mammograms are unknown in the country; a recent survey from the Sociedade Brasileira de Mastologia showed a very low coverage from 1.5% to 35% of the target population depending on the regional state [9]. Not surprisingly, reports from Brazilian physicians affirm that 80% of BC cases are brought directly to their attention by patients themselves [10]. Moreover, there is a delay between mammogram and imaging interpretation that lasts up to 30 days for 66% of screening mammograms and is more than 3 months for 20% of scans [11, 12]. Additionally, due to concerns regarding mammography quality, the Ministry of Health launched an audit programme from 2017 to 2019 to certify several institutions which perform mammography [13].

Finally, observational studies have found that the median time from BC presentation (e.g. symptoms or mammography) to diagnosis (biopsy) in Brazil is around 75–185 days, which may affect patient stage at diagnosis and survival [11, 14].

### Stage at diagnosis

The Amazona study [7, 15], Brazil’s largest BC retrospective cohort study conducted by the Grupo Brasileiro de Estudos do Câncer de Mama (GBECAM), which included 3,142 patients with a diagnosis of BC in two periods 2001 and 2005, reported differences of the stage at diagnosis depending on the type of health insurance. In the entire study population, stage distribution was: 20% stage I, 48% stage II, 28% stage III and 5% stage IV. Almost 33% of patients diagnosed in public institutions were stage III versus 16% in private institutions [7, 16]. In another cohort [16] of 1230 patients from 2012 to 2016 in a private institution in Rio de Janeiro, 79.0% had stages I or II and 16.1% were stage III. Therefore, patients from the public health system presented with a higher rate of locally advanced breast cancer (LABC) disease probably due to lower educational level and poor access to screening programmes for BC. In addition, there are also regional differences in terms of stage at diagnosis within Brazil, where patients from the states in the north and central-west regions, which have a higher rate of poverty, had a higher chance of being diagnosed with LABC [10].

### Molecular subtypes, diagnostic accuracy and genetic tests

Data from the Amazona study found that almost 70% of patients are diagnosed with estrogen receptor (ER)/progesterone receptor (PR) positive BC, around 20% with HER-2 positive (ER/PR positive or negative) and 21% with triple-negative subtype [15]. There are regional differences in the distribution of molecular subtypes. More women are diagnosed with triple-negative BC in the north and central-west than other regions, in contrast with luminal and HER-2 positive, which are more frequent in south and southeast regions [unpublished]. Other national studies showed the same distributions, which are similar to the ones described in developed countries [17].

In Brazil, there are no official quality national programmes in place aimed at the standardisation and certification of BC pathological analysis and report [10]. About 22% of fine-needle aspiration biopsy procedures obtained inadequate material for cytopathological analysis, with rates up to 38% in the poorest states [18]. Study from Salles found a concordance of only 60% in the diagnosis of 329 breast biopsies between two pathologists in a single Brazilian institution [19]. A concordance between local and central laboratory for ER and PR expression was 89.4% and 85.0%, respectively, which is similar to other countries. In terms of HER2 testing, there is a lack of standardisation in some laboratories and fluorescence *in situ* hybridisation is not available at all laboratories [10]. In general, good quality control is expected in laboratories; despite very few data being published on this matter, one study, for example, found an expressively low concordance, as low as 34%, of HER2 testing between local and central laboratories [20].

Therefore, validation and rigorous quality control measures are strongly recommended in order to avoid erroneous treatment of BC patients in Brazil.

Access to BRCA testing in Brazil is scarce, mainly due to lack of coverage from private and public health systems and few geneticists dedicated to BC or who participate in multidisciplinary meetings. A few studies [21–23] performing a profile of families at-risk for hereditary breast and ovarian cancer (HBOC) found a prevalence of 3.4% to 21.5% of patients harbouring BRCA1/BRCA2 mutations. Knowledge about the germline mutational spectrum among Brazilian HBOC patients is limited. Most importantly, the largest [24] comprehensive description of the spectrum of germline BRCA mutations in different geographical Brazilian regions showed significant molecular heterogeneity in the BRCA1 and BRCA2 genes among Brazilian carriers.

## LABC treatment in Brazil

### Surgery

There are no studies analysing the quality of BC surgery in Brazil [10]. Although surgical treatment with a breast surgeon is associated with improved outcomes, most surgeries for BC in Brazil are performed by non-specialised professionals [10], which may be associated with lower rates of breast conservation therapy and increased rates of positive margins, which may increase reoperation or the use of radiation and systemic therapy, thus increasing health care costs [25, 26]. According to official reimbursement data from Sistema Único de Saude (SUS) in 2010, 65% of therapeutic breast surgeries were mastectomies and 35% were lumpectomies [10]. The rates of mastectomy are lower in the private system (40.1%) than they are in the public system (51.7%), probably reflecting differences in BC staging at diagnosis [7].

### Radiotherapy

Radiation therapy is a key component of LABC management, with impact in local control and survival. In general, there is a shortage of radiotherapy resources in developing countries, which may impact the optimal treatment of patients with breast-conserving surgery or increase rates of mastectomy [27]. Compared to the USA, Brazil has a lower number of radiation units (9.85 versus 0.93 per million population), although that rate is comparable with other Latin American countries [28]. It is estimated that Brazil would need the double the amount of radiation equipment in the public health system to cover the whole population, as well as install the equipment in two–three states, which have none or very few machines. [29] Therefore, many patients have to travel long distances for treatment and experience delays in treatment—especially those living far from metropolitan areas [30].

### Systemic treatment

Since 2012, a Brazilian Government Federal Act has established that patients diagnosed with cancer in Brazil have access to free, contemporary treatment that must begin a maximum of 60 days after the cancer diagnosis (Law n. 12,732, Nov. 22, 2012).

Within Brazil’s public health system, which covers approximately three-fourths of the country’s population, access to HER2-targeted therapy is restricted. In the country, trastuzumab has been available for the adjuvant treatment of patients with early-stage or locally advanced disease in the public health system since 2013, almost 10 years after its approval in Europe and USA. Pertuzumab, another effective therapy in the neoadjuvant or adjuvant treatment of LABC, is still unavailable for patients under public healthcare coverage. Moreover, pertuzumab was approved only in 2018 for the treatment of metastatic BC patients. Patients with private insurance (including one-fourth of the population) do have access to all approved anti-HER2 agents trastuzumab and pertuzumab available to be used as (neo) adjuvant treatment [31].

Moreover, there are disparities in access to some types of endocrine treatment in Brazil. Although adjuvant aromatase inhibitors and tamoxifen are available for all patients, extended endocrine treatment beyond 5 years is not covered in the public health system, losing the benefit of this therapeutic strategy in selected patients. [32–34] A recent prospective registry (AMAZONA III) conducted by GBECAM and the Latin American Cooperative Oncology Group shows a high prevalence of young women (8.4% ≤ 35 years and 34.8% with 36–50 years) diagnosed with BC from 2016 to 2018 in Brazil (unpublished data). However, pre-menopausal women with BC have limited access to ovarian function suppression (OFS) drugs (e.g. gonadotropin-releasing hormone agonists) to preserve fertility or to be used in combination with adjuvant endocrine treatment [35–37]. Therefore, despite the established benefit of OFS in terms of disease-free and overall survival demonstrated in the SOFT and TEXT trials [38], young women with LABC who would benefit more with this regimen are at high risk of poor outcome in Brazil.

The process of drug approval in Brazil by the Brazilian regulatory agency Agencia Nacional de Vigilancia Sanitaria doesn’t guarantee access to all patients, approved drugs must be provided by the private health insurance and another step of approval by the Comissão Nacional de Incorporação de Tecnologias do SUS provides the drugs for the public system, so several cancer drugs beyond those for BC are not available to cancer patients.

## BC research

### Access to clinical trials in Brazil

Clinical research is an opportunity for optimal treatment and innovative therapies for BC patients, as well as professional development for investigators and qualification for institutions. In a survey, 94% of oncologists practicing in Latin America reported that there was insufficient clinical-epidemiological research on BC in their country. The main barriers are lack of time and financial support; therefore, clinical trials in Brazil and Latin America are mainly pharmaceutical sponsored trials and less than 1% are academic studies. [39]

Although further investment in clinical research is needed throughout Latin America, Brazil is already a leader in this area and many Brazilian clinical trial sites have higher enrolment rates than do those in the USA or Europe [40], being attractive for international trials. One reason for these high accrual rates is the disparity in access to good standard therapies in many public services, making the participation in clinical trials an attractive option for patients [10].

Compared with other Latin American countries, the number of BC trials open in Brazil is higher; however, this is less than 5% of all studies being conducted worldwide.

The average time for regulatory approval of a research protocol in Brazil is 6–7.5 months, compared with 2–3 months in the USA [41]. This has an important effect in the competitiveness of Brazil to attract more studies, especially phase I and II trials.

These facts are reflected in a low number of publications about BC from Brazil. In the last 5 years, only 146 articles were published in PubMed indexed journals and compared with other Latin American countries, Brazil has a greater scientific production.

## Conclusion

A high proportion of patients are diagnosed with LABC in Brazil, mainly due to limited coverage of screening programmes. A disparity in the access to optimal treatment is evident between public and private health systems which impacts patient outcomes. Clinical research is an opportunity for patients, institutions and investigators and therefore, should be facilitated through a better regulatory environment. In a country facing a trend of increasing BC incidence over the upcoming years, it is critical to improve BC screening and incorporate new medicines and devices into the public health system to control the LABC burden.

## Conflicts of interest

The authors declare that they do not have any potential conflict of interest regarding this review article.

## Funding statement

The authors did not have any funding source related to this review article.
